# Professor Carl Sauer

**Published:** 1892-07

**Authors:** 


### PROFESSOR CARL SAUER.

The death of Professor Sauer, on March 17, 1892, at Berlin, will leave a blank in dental circles in Germany not easily filled. His work has been unceasing for many years, indeed he may be regarded almost as the father of modern dental thought and practice in that country.

For several years he was honored with the presidency of the Central Association of German Dentists. His recognized ability as a metallurgist, and in general mechanical dentistry, led to his selection by the government for a professorship in the Institute of Dentistry of the University of Berlin. This position he held up to the period of his death.

His name has long been a familiar one on this side of the Atlantic, and there will be many who will read of his death with feeling and sympathy for those immediately affected by his loss. The world has been made richer by his life, and in his departure he leaves dentistry in his own land advanced to a position which could not have been anticipated twenty years ago. For this success Professor Carl Sauer should be accredited a large share of honor.

